# Implementation of support meetings for patients undergoing outpatient chemotherapy by a multidisciplinary cancer team

**DOI:** 10.20407/fmj.2024-005

**Published:** 2024-10-31

**Authors:** Miho Jinno, Misako Dai, Kaori Ito, Yosuke Ando, Seira Toyosato-Nishibe, Maki Akiyoshi, Sachie Noda, Hidezo Matsuda, Naho Tsujii, Miho Zennami, Masami Yamamura, Yoko Katagata, Akemi Ito, Aki Takai, Shigeki Yamada, Kenji Kawada, Junko Sugama, Keiko Mano

**Affiliations:** 1 Division of Nursing, Fujita Health University Hospital, Toyoake, Aichi, Japan; 2 Research Center for Implementation Nursing Science Initiative, Research Promotion Headquarters, Fujita Health University, Toyoake, Aichi, Japan; 3 Department of Pharmacotherapeutics and Informatics, Fujita Health University, School of Medicine, Toyoake, Aichi, Japan; 4 Faculty of Pharmacy, Meijo University, Nagoya, Aichi, Japan; 5 Department of Pharmacy, Fujita Health University Hospital, Toyoake, Aichi, Japan; 6 Department of Nutrition, Fujita Health University Hospital, Toyoake, Aichi, Japan; 7 Department of Medical Oncology, Fujita Health University, School of Medicine, Toyoake, Aichi, Japan

**Keywords:** Outpatient chemotherapy, Communication, Multidisciplinary team, Quality of health care

## Abstract

**Background:**

Outpatient chemotherapy is a standard treatment for cancer. In nursing care for outpatients, it is important to enhance patients’ self-efficacy. Vicarious experiences that can be gained through interactions with other patients with cancer can be useful for achieving this. While inpatients can gain vicarious experiences through their hospital stay, outpatients typically have fewer opportunities to do so.

**Aims:**

This report aimed to examine the results of implementation of support meetings for patients receiving outpatient chemotherapy.

**Methods:**

Starting in April 2019, support meetings were held once a month for outpatients on Thursdays from 14:00 to 16:00 in a hospital conference room. Medical professionals designed the programme of support meetings to allow patients to interact with each other and engage in vicarious experiences. At each meeting, satisfaction regarding the support meeting content was evaluated by self-administered questionnaire. Moreover, the nurse asked all participants to talk about their interactions, and recorded and extracted narratives about vicarious experiences.

**Findings:**

The 32 participants had a median age (interquartile range) of 63.5 years (55–70 years). There were 26 females (81.2%). The median satisfaction scores ranged from 2.9 to 4 for the content of each meeting. Patients talked about the value of learning from the experiences of other patients and the easing of loneliness.

**Conclusion:**

The results suggested that support meetings can provide vicarious experiences for patients receiving outpatient chemotherapy.

## Background

The number of cancer cases has increased worldwide in recent years,^1^ and cancer treatment has become a major component of healthcare. Outpatient chemotherapy is a standard treatment for cancer, allowing patients to continue treatment while living their daily lives, and providing an effective cure in some cases.^2^ However, patients may confront various problems while undergoing chemotherapy in their daily lives. Complex problems, such as the appearance of various physical symptoms of cancer, side effects of chemotherapy, psychological changes, and social problems^3–5^ significantly impact patients’ quality of life.^6^ Therefore, providing support to cancer patients undergoing outpatient chemotherapy to help them balance their daily lives and treatment is important for improving healthcare quality related to cancer management.

Clinical care provision systems that can provide consultation and responses to problem-solving for physical, psychological, and social effects is essential for supporting continued outpatient chemotherapy in cancer patients. Emphasis has been placed on psychological support that aims to improve self-efficacy to maintain patients’ will to continue treatment and their motivation to consult medical professionals as soon as a problem occurs or is about to occur.^7,8^

Bandura proposed the concept of self-efficacy in the framework of social learning theory.^9,10^ Four factors can determine self-efficacy on the basis of information sources: the accomplishment of performance behaviours, vicarious experience, verbal persuasion, and physiological state.^9^ Vicarious experiences of learning by watching others deal with challenging situations can encourage patients, particularly when they meet others who have the same disease and have completed treatment, or who have recovered after being discharged from hospital.^10^

Outpatient treatment conventionally involves medical professionals listening to patients’ stories and providing opportunities for interviews between professionals and patients, which are examples of verbal persuasion within self-efficacy. However, while inpatients can gain vicarious experiences through their hospital stay, outpatients typically have fewer opportunities to do so. Therefore, in the current report, a team of cancer management experts regularly planned and conducted support meetings where patients receiving outpatient chemotherapy could communicate with each other and engage in vicarious experiences by seeing and listening to others with similar experiences.

This practical report describes the results of implementation of support meetings for patients receiving outpatient chemotherapy.

## Methods

### Setting

The support meetings were conducted at a 1,376-bed university hospital’s outpatient chemotherapy centre in Aichi Prefecture, Japan. The centre conducts 20,000 chemotherapy treatments annually.

### Support meeting management team

In Japan, The Ministry of Health, Labour and Welfare directs professionals who belong to the cancer team. The management team comprised: one oncology specialist doctor (Japanese Society of Medical Oncology-certified); two certified nursing managers (Japanese Nursing Association-certified); one wound, ostomy and continence nurse; four cancer chemotherapy nursing-certified nurses; one medical pharmacy-specialised pharmacist (Japanese Society of Pharmaceutical Health Care and Sciences-certified); six cancer specialist pharmacists (Japanese Society of Pharmaceutical Health Care and Sciences-certified); and one registered dietitian specialising in cancer pathological nutrition (Japan Society of Metabolism and Clinical Nutrition-certified).

### Intervention

Starting in April 2019, support meetings were held once a month for outpatients on Thursdays from 14:00 to 16:00 in the hospital conference room.

Meetings comprised lectures and relaxation activities. Because they were targeted to patients undergoing cancer treatment, meetings were planned and overseen by members of the management team. Lectures were delivered by a medical professional who specialised in supportive care and financial support for cancer side effects. Relaxation activities involved tea parties, seasonal events unique to Japan, and massages. Medical professionals designed the programme of support meeting to allow patients to interact with each other and engage in vicarious experiences. The content of each meeting was confirmed by a certified nursing manager. Communication was coordinated and operational support was provided within the hospital.

Patients were free to participate in each meeting. Those who responded to flyers left in the outpatient chemotherapy centre were recruited. At least three patients, the oncology specialist, the certified cancer nurse, and other members were present in each support meeting.

### Outcomes of intervention

Patients provided responses regarding their satisfaction with the content of each meeting using a questionnaire distributed at the end of each healthcare community session; answers were rated on a five-point Likert scale, ranging from 0=“not satisfied at all” to 5=“very satisfied.”

The nurse asked all participants to talk about their interactions, and recorded the patients’ narrative in a daily report.

### Analysis

The level of satisfaction with the content of the sessions was calculated as the median (interquartile range) of participants’ monthly satisfaction evaluations. The median (interquartile range) of the satisfaction evaluation on the basis of the total number of participants was also calculated.

From the daily report on their narratives, the researchers extracted a description of vicarious experiences.

Descriptive statistics are shown as N (%) for categorical variables and median (interquartile range) for continuous variables.

### Ethical considerations

Study approval was obtained from the Medical Ethics Review Committee of Fujita Health University (HM21-474). A written explanation was given to each patient, and written consent to participate was obtained.

## Results

### Participants

The 32 participants had a median age (interquartile range) of 63.5 years (55–70 years), and 26 were female (81.2%). The most common cancer type was breast cancer (13 patients, 40.6%), followed by lung cancer (four patients, 12.5%) and colorectal cancer (four patients, 12.5%). Cases of uterine, ovarian, stomach, and kidney cancers were also observed. Stage II (17 patients, 53.1%) was the most common cancer stage, followed by stage IV (37.5%), stage III (two patients, 6.3%), and stage I (one patient). The performance status was 0 for all patients. Three patients (8.8%) were receiving palliative care (Table 1).

### Number of participants for each support meeting and satisfaction scores

Table 2 shows the number of participants and their satisfaction scores. There were 3–12 participants in each meeting, and median scores were distributed between 3 and 4. The median satisfaction scores were 3.5–4 for meetings that included both lectures and relaxation, 4 for meetings with only lectures, and 3–4 for meetings with only relaxation.

Table 3 shows the satisfaction score for the number of meetings attended by patient. It was most common for patients to participate once, accounting for 19 patients (55.9%); the median satisfaction score of these patients was 4. Fewer than two patients participated in three or more meetings, and their median satisfaction score ranged from 2.9 to 3.8.

### Vicarious experiences for enhancing self-efficacy

Table 4 summarises narratives of the participating patients. Patients talked about the value of learning from the experiences of other patients and the easing of loneliness.

*“Meeting other patients who experienced the same disease was very encouraging. I didn’t need to participate in every meeting; I only participated when I felt anxious or had concerns.”*

*“When I became sick, I thought ‘why me’, and I really struggled. Participating in this session allowed me to realise that it’s not just me, and that other patients are also doing their best.”*

## Discussion

A total of 10 support meetings were held for patients undergoing treatment in the outpatient chemotherapy centre. Patients’ median satisfaction score regarding the content was 4 points (satisfied) for 7 out of 10 meetings. There were no large differences in satisfaction between different combinations of lectures and relaxation activities. Additionally, the median satisfaction score was 4 points even for participants who participated in only one meeting. This finding suggests that such healthcare community activities could be easily conducted among patients undergoing outpatient chemotherapy in the future.

The present study examined an initiative that was designed to support patients to improve self-efficacy by engaging in vicarious experiences. Patients who participated once or twice said, “Meeting other patients who experienced the same disease was very encouraging. I didn’t need to participate in every meeting; I only participated when I felt anxious or had concerns.” This response suggests that patients had vicarious experiences.

The current study examined in-person support meetings. In the future, however, the use of an online format could be considered as a countermeasure against infectious diseases, such as COVID-19. Telephone, chat, and online consultation systems are currently widely used to provide remote support to cancer patients.^11,12^ However, these services constitute communication between medical professionals and patients, and do not provide vicarious experiences involving joint participation of multiple patients and experts, which was the purpose of the support meetings in the present study. The development of appropriate online methods could be useful for promoting the dissemination of support meetings and providing support to patients facing various problems in daily life while undergoing cancer treatment.^13^ The current study is expected to improve the quality of healthcare in the cancer management field.

The current study involved several limitations that should be acknowledged. First, the study did not use a randomised controlled trial design. Thus, we were unable to establish the effectiveness of the support meetings. Second, the satisfaction scale was administered by the medical professionals who planned and operated the support meetings, and who were also closely involved in patients’ cancer treatment. Thus, the support meeting participants (i.e., patients with cancer) may have been more likely to respond in a way that they expected medical professionals to appreciate. Third, the sample size was relatively small (32 patients). The sample size was limited because the support meetings were started in April 2019, and had to be cancelled in February 2020 because of the COVID-19 pandemic. Furthermore, meetings that were held regularly on the fourth Thursday of each month coincided with the closure of the mammary surgery outpatient centre, which resulted in a larger number of female than male participants.

## Conclusion

The current results suggested that patients undergoing cancer outpatient chemotherapy were satisfied with the content of the support meetings. In these meetings, we found that patients were able to communicate with each other and engage in vicarious experiences. Future studies should develop approaches for implementing support meetings, in-person and online, to support cancer patients undergoing chemotherapy.

## Figures and Tables

**Table1:** Patients’ characteristics N=32

Variables	
Age (years)	63.5 (55–70)
Gender	
Male	6 (18.8)
Female	26 (81.2)
Cancer type	
Breast cancer	13 (40.6)
Colorectal cancer	4 (12.5)
Lung cancer	4 (12.5)
Ovarian cancer	2 (6.3)
Prostate cancer	1 (3.1)
Uterine cancer	1 (3.1)
Stomach cancer	1 (3.1)
Liver cancer	1 (3.1)
Others	5 (15.6)
Stage of cancer	
I	1 (3.1)
II	17 (53.1)
III	2 (6.3)
IV	12 (37.5)
Performance status	
0	32 (100.0)
Receiving palliative care	
Yes	3 (8.8)

Continuous variable: Median (interquartile range) Categorical variable: N (%)

**Table2:** Health care community contents and participant satisfaction

Session year/month	Lecture theme	Relaxation activities	Number of operating staff	Number of patients	Satisfaction score (median)
2019 April	How to choose a wig	Tea party	6	8	4
	Medical makeup
May		Tea party	7	7	3
June		Seasonal event	8	7	4
		Tea party	
July		Seasonal event	10	3	4
August	Interactions between anticancer drugs and food	Tea party	10	6	3.5
September	Utilisation of social resources for cancer patients	Tea party	9	8	4
	Employment support
October		Hand massage	8	8	4
		Tea party	
November	Dietary management during cancer treatment		9	12	4
	Preparing meals for side effects
December	Medical narcotics		9	8	4
2020 January		Hand massage	7	6	3
		Tea party			

**Table3:** Number of attendance and patients’ satisfaction

Number of meetings attended by each patient	N (%)	Satisfaction Median (IQR)
1	19 (55.9)	4 (3–4)
2	8 (23.5)	3.5 (3.3–4)
3	2 (5.9)	3.3 (3.3–3.3)
4	0 (0.0)	—
5	2 (5.9)	3.5 (3–4)
6	2 (5.9)	3.8 (3.7–4)
7	0 (0.0)	—
8	1 (2.9)	2.9 (2.9–2.9)

IQR: interquartile range

**Table4:** Patients’ vicarious experiences at support meetings

Vicarious experience
• Encounters with other patients who experienced the same disease were very valuable and encouraging.
• When I became sick, I thought ‘why me’, and I really struggled. Participating in this session allowed me to realise that it’s not just me and that other patients are also doing their best.
• I want to meet more cancer patients and hear their stories.
• I thought that there’s no way that someone who has not had cancer would understand my feelings, and I was unable to consult with anybody. Every word of the stories of other patients had weight, and they resonated with me.
• Everybody has their own struggles and sadness, but I was able to start thinking, ‘It’s not just me.’ I felt like I could finally hold my head up high and live. I was able to feel that way with just one session.
• I was able to laugh for the first time in a while. I could let my guard down.
• I want to participate as often as possible, meet many other patients, and share this feeling. If my experience encourages other patients, then I want to tell my story.
